# Glycan-Based Near-infrared Fluorescent (NIRF) Imaging of Gastrointestinal Tumors: a Preclinical Proof-of-Concept *In Vivo* Study

**DOI:** 10.1007/s11307-020-01522-8

**Published:** 2020-08-11

**Authors:** Ruben D. Houvast, Victor M. Baart, Shadhvi S. Bhairosingh, Robert A. Cordfunke, Jia Xin Chua, Mireille Vankemmelbeke, Tina Parsons, Peter J. K. Kuppen, Lindy G. Durrant, Alexander L. Vahrmeijer, Cornelis F. M. Sier

**Affiliations:** 1 Department of Surgery, Leiden University Medical Centre, Albinusdreef 2, 2333 ZA Leiden, the Netherlands; 2 Department of Immunohematology and Blood Transfusion, Leiden University Medical Centre, Leiden, the Netherlands; 3 Scancell Limited, University of Nottingham Biodiscovery Institute, University Park, Nottingham, UK; 4 Division of Cancer and Stem Cells, School of Medicine, University of Nottingham Biodiscovery Institute, University Park, Nottingham, UK; 5 Percuros BV, Leiden, the Netherlands

**Keywords:** Carbohydrates, Lewis glycans, Aberrant glycosylation, Monoclonal antibody, Fluorescence-guided surgery

## Abstract

**Purpose:**

Aberrantly expressed glycans in cancer are of particular interest for tumor targeting. This proof-of-concept *in vivo* study aims to validate the use of aberrant Lewis glycans as target for antibody-based, real-time imaging of gastrointestinal cancers.

**Procedures:**

Immunohistochemical (IHC) staining with monoclonal antibody FG88.2, targeting Lewis^a/c/x^, was performed on gastrointestinal tumors and their healthy counterparts. Then, FG88.2 and its chimeric human/mouse variant CH88.2 were conjugated with near-infrared fluorescent (NIRF) IRDye 800CW for real-time imaging. Specific binding was evaluated *in vitro* on human gastrointestinal cancer cell lines with cell-based plate assays, flow cytometry, and immune-fluorescence microscopy. Subsequently, mice bearing human colon and pancreatic subcutaneous tumors were imaged *in vivo* after intravenous administration of 1 nmol (150 μg) CH88.2-800CW with the clinical Artemis NIRF imaging system using the Pearl Trilogy small animal imager as reference. One week post-injection of the tracer, tumors and organs were resected and tracer uptake was analyzed *ex vivo*.

**Results:**

IHC analysis showed strong FG88.2 staining on colonic, gastric, and pancreatic tumors, while staining on their normal tissue counterparts was limited. Next, human cancer cell lines HT-29 (colon) and BxPC-3 and PANC-1 (both pancreatic) were identified as respectively high, moderate, and low Lewis^a/c/x^-expressing. Using the clinical NIRF camera system for tumor-bearing mice, a mean tumor-to-background ratio (TBR) of 2.2 ± 0.3 (Pearl: 3.1 ± 0.8) was observed in the HT-29 tumors and a TBR of 1.8 ± 0.3 (Pearl: 1.9 ± 0.5) was achieved in the moderate expression BxPC-3 model. In both models, tumors could be adequately localized and delineated by NIRF for up to 1 week. *Ex vivo* analysis confirmed full tumor penetration of the tracer and low fluorescence signals in other organs.

**Conclusions:**

Using a novel chimeric Lewis^a/c/x^-targeting tracer in combination with a clinical NIRF imager, we demonstrate the potential of targeting Lewis glycans for fluorescence-guided surgery of gastrointestinal tumors.

**Electronic supplementary material:**

The online version of this article (10.1007/s11307-020-01522-8) contains supplementary material, which is available to authorized users.

## Introduction

Recent advances in surgical techniques, like laparoscopy and robotics, have reduced the ability for surgeons to directly palpate the surgical field, the second-best sense for recognition of abnormalities after visualization [1]. Consequently, various techniques and technologies have been introduced to aid surgeons in identifying key structures. Targeted image-guided surgery, based on near-infrared fluorescent (NIRF) light, has been shown to be a valuable tool for distinguishing malignant from healthy tissue during oncologic surgery [2]. The key elements of this technique include an efficient tracer-target combination and a dedicated NIRF camera system. Currently, the major challenge in molecular imaging remains the identification of the most suitable target for the tumor of choice. Targeted imaging tracers ideally detect all tumor cells, not only within the primary tumor but also in lymph nodes and distant metastasis and visually occult lesions. The potential of established tumor-specific proteins, such as carcinoembryonic antigen (CEA), epidermal growth factor receptor (EGFR), epithelial cell adhesion molecule (EpCAM), human epidermal growth factor receptor 2 (HER2), vascular endothelial growth factor (VEGF), and several integrins, as targets for tumor imaging has been successfully demonstrated in both preclinical and clinical settings [2–10]. Most target/tracer combinations appear to have shortcomings, such as excessive interaction with normal tissues, serum instability, or an unsuitable clearance profile, resulting in lack of tumor/background contrast. Therefore, a quest for novel, less conventional imaging targets seems essential, if not indispensable.

Aberrant glycosylation of proteins and lipids is considered a hallmark of cancer [11, 12]. During oncogenesis, immature mucin-type *O*-glycans, such as sialyl-Thomsen-Nouvelle (sTn), and fucosylated glycan antigens, such as sialyl-Lewis^a^ (sLe^a^/CA19.9) and sialyl-Lewis^x^ (sLe^x^/CD15s), are overexpressed on the cell membrane of cancer cells. Some of these antigens, like sLe^a^ and sLe^x^, seem heavily involved in tumor progression, invasion, and metastasis, whereas their role in healthy tissue is minimal [13–15]. Therefore, targeting of tumor-associated glycans offers opportunities not only for therapy but also for molecular imaging. Originating from genetic dysregulation of the enzymes responsible for glycan synthesis, glycan expression is not limited to a single protein [16]. Hence, tracers against tumor-associated glycans will target multiple tumor-associated proteins and lipids simultaneously and may provide a broader tumor-targeting strategy than targeting each tumor marking protein separately. Because glycans are less immunogenic than proteins, the number of specific IgG antibodies against glycans is still limited [17]. Recently Chua et al. developed the novel anti-Le^c^Le^x^, di-Le^a^, Le^a^Le^x^, and Le^a^ IgG antibody FG88.2, which showed specific immunohistochemical staining on 81 % of pancreatic, 71 % of colorectal, 54 % of gastric, 23 % of non-small cell lung, and 31 % of ovarian tumor tissues, along with a restricted binding to normal tissues [18]. Subsequently a chimeric (mouse/human) variant was developed, termed CH88.2. This variant is composed of a human Fc region but contains the same mouse-derived antigen binding region as FG88.2, essentially preserving its target specificity. Given the expression of its glycotarget, antibody CH88.2 conjugated with an NIRF dye might constitute a valuable pan-carcinoma tracer for fluorescence-guided surgery (FGS).

In this study, we validate the concept of glycan-based real-time imaging of gastrointestinal tumors by using CH88.2 conjugated with NIR fluorophore IRDye800CW. Specific binding of the antibodies was confirmed on human gastrointestinal tissues and a range of gastrointestinal cell lines. The tracer specificity was evaluated *in vivo* using subcutaneous mouse models of gastrointestinal cancers. Using a chimeric antibody in combination with the clinical equivalents of a NIR system, we might pave the way for a rapid clinical translation, not only for this particular tracer but also for the concept of imaging of cancers using glycan-targeting tracers.

## Materials and Methods

### Monoclonal Antibodies

Anti-Le^c^Le^x^, di-Le^a^, Le^a^Le^x^, and Le^a^ mouse FG88.2 (mIgG_3_) and its chimeric derivate CH88.2 (hIgG_1_) were supplied by professor Lindy Durrant (Scancell Ltd, UK).

### Monoclonal Antibody Conjugation

Mouse FG88.2 and CH88.2 were covalently conjugated with NIR fluorochrome IRDye800CW *via* N-hydroxysuccinimide (NHS)-ester chemistry against primary amines until a degree of labeling (DOL) between 1 and 1.5 was reached, following the manufacturer’s protocol (LI-COR, Lincoln, NE, Nebraska). DOLs were estimated by the supplied mathematical formula and confirmed by Maldi-TOF analyses using a Microflex (Bruker, Billerica, MA, USA) and sinnapinic acid as matrix.

### Immunohistochemistry

Formalin-fixed, paraffin-embedded tissue blocks from colon tumors (*n* = 4), gastric tumors (*n* = 8), pancreatic tumors (*n* = 10), and pancreatitis (*n* = 2), particularly selected for the presence of healthy appearing adjacent tissue, were obtained from the Pathology department of the Leiden University Medical Center (LUMC). Immunohistochemical staining was performed on 4-μm-thick sections on glass slides. The sections were deparaffinized in xylene for 15 min, rehydrated in a series of ethanol dilutions, and rinsed in demineralized water. Next, endogenous peroxidase was blocked with 0.3 % hydrogen peroxide in demineralized water. Antigen retrieval was performed by heating sections to 95 °C for 10 min in EnVision Flex Target Retrieval Solution (pH 6.0) using PT Link (Dako, Glostrup, Denmark). After cooling for 5 min in PBS (phosphate-buffered saline, pH 7.4), sections were incubated overnight in a humidified chamber at room temperature with 150 μl primary mouse FG88.2 antibody (0.19 μg/ml). Sections were washed three times in PBS for 5 min and incubated with secondary goat anti-mouse EnVision antibody (Dako, K4001) for 30 min. After secondary incubation and additional washing, sections were incubated with DAB+ substrate buffer (Dako) for 10 min. Sections were counterstained with Mayer’s hematoxylin solution (Sigma-Aldrich, Saint Louis, MO, USA), dehydrated in an incubator for 1 h at 37 °C and mounted with Pertex (Leica Microsystems, Wetzlar, Germany). To exclude nonspecific staining, a negative (PBS) and conjugate control (only secondary antibody) were included. Slides were examined under a Zeiss AxioSkop 20 light microscope (Carl Zeiss, Jena, Germany).

### Human Cancer Cell Lines

Cell lines KATO III (signet ring diffuse cell type gastric carcinoma), HT-29, DLD-1, COLO 205, HCT-15 (colon carcinoma), BxPC-3(_luc2), PANC-1, MIA PaCa-2 (pancreatic carcinoma), and CHO (Chinese hamster ovary) were obtained from ATCC, except for BxPC-3_luc2 which was purchased from PerkinElmer (Waltham, MA, USA). KATO III, HT-29, DLD-1, COLO 205, HCT-15, and BxPC-3(_luc2) cells were cultured in RPMI 1640 cell culture medium (Gibco, Invitrogen, Carlsbad, CA, USA). PANC-1, MIA Paca-2, and CHO cells were cultured in DMEM + GlutaMAX™ cell culture medium (Gibco, Invitrogen). Both media were supplemented by l-glutamine, 25 mM HEPES, 10 % fetal bovine serum (FBS; Hyclone, Thermo Scientific, Rockford, Il, USA), and penicillin/streptomycin (both 100 IU/ml; Invitrogen). Absence of mycoplasma was confirmed using polymerase chain reaction. Cells were grown to 90 % confluence in a humidified incubator at 37 °C (5 % CO_2_) and detached with trypsin/EDTA. Viability was assessed using Trypan Blue staining in 0.4 % solution (Invitrogen).

### Cell-Based Plate Assay

Binding of FG88.2-800CW was evaluated on gastrointestinal cancer cell lines KATO III, HT-29, DLD-1, COLO 205, HCT-15, BxPC-3, PANC-1, and MIA PaCa-2 using a plate assay with CHO as reference cell line. Cells were grown in a 96-well plate (Corning Costar Inc., Cambridge, MA, USA) at 20,000 cells/well in 100 μl of complete medium until 90 % confluence. Thereafter, cells were incubated with FG88.2-800CW at 10, 5, 2.5, or 1.25 μg/ml for 1 h at 37 °C. After washing twice with medium, fluorescence signal was measured using the Odyssey NIR imaging system (LI-COR Biosciences, 800-nm channel, intensity 10). The 800-nm fluorescence signal was corrected for the number of cells using a nuclear staining. Briefly, cells were fixated and permeabilized with acetone and methanol in a 40/60 mixture for 10 min. After washing, cells were incubated with TO-PRO3 (1/2000, Invitrogen) for 5 min at room temperature, washed, and scanned with the Odyssey NIR imaging system (700-nm channel, intensity 9). The mean fluorescence intensity (MFI) was calculated by dividing the 800CW fluorescence signal by the nuclear 700-nm signal and multiplying the number by 100. Measurements were performed in triplicate.

### Flow Cytometry

After detachment and viability assessment, cells were adjusted to 0.5 × 10^6^ cells/tube in PBS/BSA (PBS/bovine serum albumin) (0.5 %) and incubated with 100 μl FG88.2 antibody (5 μg/ml). Next, cells were washed twice in PBS/BSA 0.5 % and incubated with secondary AF488-labeled goat anti-mouse (A21121, Thermo Scientific, 1/800) or AF647-labeled goat anti-mouse (A21241, Thermo Scientific, 1/800) for 30 min. After washing twice with PBS with 0.5 % BSA, cells were resuspended in 400 μl PBS/BSA containing propidium iodide (1/4000) and measured on a LSRII flow cytometer (BD Biosciences, Franklin Lanes, NJ, USA; 1.0 × 10^5^ live cells per tube) using the 530/30 laser for measuring AF488 signals and 695/40 laser for measuring PI or AF647 signals. All incubation steps were done on ice, avoiding exposure to light.

### Chamber Slides

After detachment and viability assessment, cells were placed in an 8-well Nunc™ Lab-Tek™ II Chamber Slide (0.7 cm^2^/well, Thermo Scientific) at 50,000 cells/well. When approximately 90 % confluence was reached, medium was removed and cells were washed twice in PBS for 5 min. Cells were subsequently fixated with 1 % paraformaldehyde for 10 min at room temperature. After washing twice in PBS for 5 min, cells were incubated with respectively primary CH88.2-800CW, secondary polyclonal rabbit anti-human antibody (A0423, 10 μg/ml; Agilent, Santa Clara, CA, USA), and tertiary goat anti-rabbit F(ab′)2-AF488 (Thermo Scientific, A11070, 1/800) for 30 min, with two wash steps (PBS, 5 min) in between incubations. After additional washing with PBS and demineralized water, slides were dried. Next, the plastic chambers were removed and cell nuclei were stained using ProLong Gold containing DAPI (Thermo Scientific). Antibody binding was analyzed using a DM5500 B fluorescence microscope (Leica Microsystems)) with filter cube A (excitation 340–380, long pass emission 425; exposure time 0.05 s) for visualizing DAPI signals and filter cubes I3 (excitation 450–490, long pass emission 515; exposure time 0.40 s) and CY7 (excitation 710/75, emission 810/90; exposure time 0.70 s) for visualizing AF488 and 800CW fluorescence signals, respectively.

### Animal Models

Mice were kept at the Central Animal Facility of the LUMC, which houses animals per EU Recommendation 2007-526-EC under specific pathogen-free conditions [19]. For all animal handlings, local standard operating procedures were followed. Six- to eight-week-old female BALB/c-Nude (CAnN.Cg-Foxn1nu/Crl) mice (Charles River Laboratories, Wilmington, MA, USA) were subcutaneously inoculated on 4 spots on the back with either HT-29 or BxPC-3_luc2 cells (5.0 × 10^5^ cells/spot; 3 mice per group). Tumor growth was monitored by a digital caliper. Tumors of 50 mm^3^ were considered large enough for imaging. The local animal welfare body of the LUMC reviewed and approved all animal studies. Animals received humane care in compliance with the Code of Practice Animal Experiments in Cancer Research.

### *In Vivo* NIRF Imaging

The tail vein of the mice was injected intravenously with 1 nmol (150 μg) CH88.2-800CW. The mice were imaged at 4 h, 24 h, 48 h, 72 h, 96 h, 120 h, 148 h, and 168 h post-injection, using the clinical Artemis NIR Imaging System (Quest Medical Imaging b.v., Middenmeer, The Netherlands; hereafter referred to as “Artemis”) using the more sensitive but preclinical Pearl Trilogy Small Animal Imaging System (LI-COR Biosciences; hereafter referred to as “Pearl”) as a reference. Mice were kept under 2–4 % isoflurane anesthesia during imaging. After the last measurement, mice were sacrificed and the organs were removed and imaged *ex vivo* using the Pearl.

### NIRF Imaging Analysis

MFIs were extracted from images by marking a region of interest on the macroscopic tumor (tumor signal) and on the adjacent skin (background signal) using Spectrum Capture Suite (Quest Medical Imaging b.v.) and ImageJ version 5.2p for Artemis images [20] and Image Studio version 5.2 (LI-COR Biosciences) for Pearl images. Tumor-to-background ratios (TBRs) were calculated *via* the following formula: TBR = MFI tumor/MFI background. For biodistribution analysis, mean organ MFIs were calculated in Image Studio by drawing a ROI over the designated organ. Tumor-to-organ ratios were calculated by dividing the tumor MFI by the mean organ MFI of the same mouse (*n* = 3 for both HT-29 and BxPC-3 mice).

### Histological Analysis

After 1 week (168 h), tumors were resected and incubated in 4 % paraformaldehyde which was replaced by 70 % ethanol the next day. Subsequently, tumors were treated with a standard dehydration sequence (ethanol and xylene) and imbedded in paraffin.

For *ex vivo* imaging and staining, 4-μm-thick tissue sections were deparaffinized in xylene for 15 min and fluorescence imaging was performed using the Odyssey CLx NIR imaging system on the 800-nm channel. Sections were rehydrated as described in the “Immunohistochemistry” section and subsequently stained with standard hematoxylin-eosin staining.

### Statistical Analyses

Statistical analyses and graph generation were performed with GraphPad Prism (version 8.01, GraphPad Software Inc., La Jolla, CA, USA). Differences between mean TBRs and tumor/background MFIs for different time points were compared *via* one-way ANOVA. Correction for multiple comparisons was done using the Holm-Sidak method. Differences in biodistribution between HT-29 and BxPC-3_luc2 mice were calculated using independent samples *t* tests. Differences with a *P* value smaller than 0.05 were regarded significant (NS: not significant; *: *P* ≤ 0.05; **: *P* ≤ 0.01; ***: *P* ≤ 0.001).

## Results

### Immunohistochemistry

**Fig. 1.**
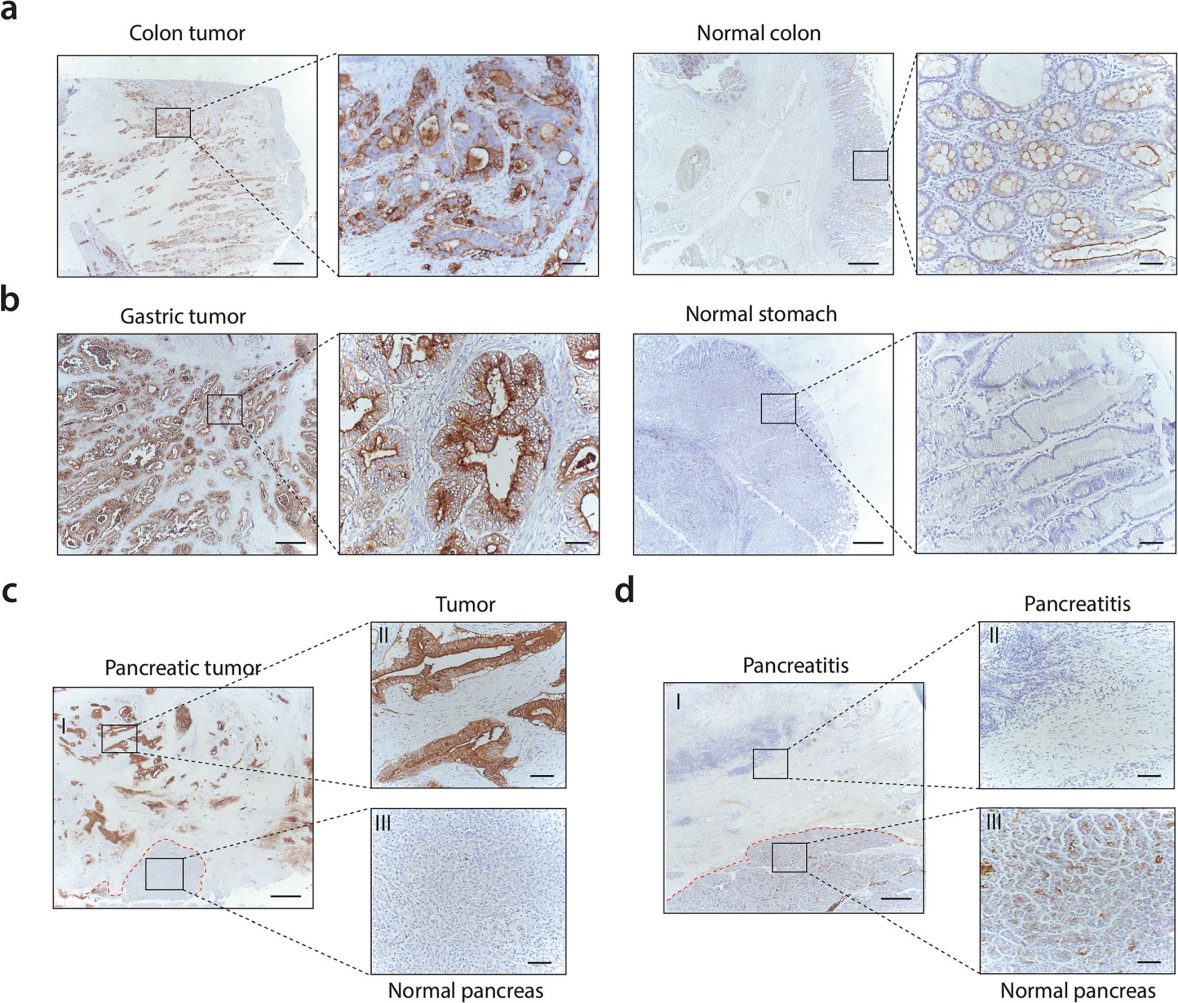
**a** Immunohistochemical FG88.2 staining in a colon tumor and in normal colonic crypts. **b** FG88.2 staining in a gastric tumor and in normal gastric glands. **c**, **d** FG88.2 staining in pancreatic tumor tissue and pancreatitis tissue (and normal pancreatic tissue derived from the same patient (III)). Red-dotted lines represent the tumor (**c**) or pancreatitis-normal pancreatic tissue border (**d**). Overview images are taken at × 50 magnification and inserts at × 200 magnification. Scale bars represent 500 μm and 100 μm, respectively. Scale bars represent 500 μm and 100 μm for overview and insert images, respectively.

Immunohistochemical analysis showed FG88.2 staining in 1 out of 4 colon tumors (Fig. 1a), 4 out of 8 gastric tumors (Fig. 1b), and 7 out of 10 pancreatic tumors (Fig. 1c). FG88.2 was mainly located on the basolateral and apical membrane of cancer cells, and also some staining in cytoplasm was observed. Stromal cells did not stain. FG88.2 on normal colon was mainly located near the apical membrane of epithelial cells and was low to moderate in normal colon (Fig. 1a), negative to moderate in normal stomach glands (Fig. 1b), and negative to weak in healthy pancreatic acini and ducts (Fig. 1c). Very limited FG88.2 staining was found on both pancreatitis tissue samples (Fig. 1d).

### Binding Specificity

**Fig. 2.**
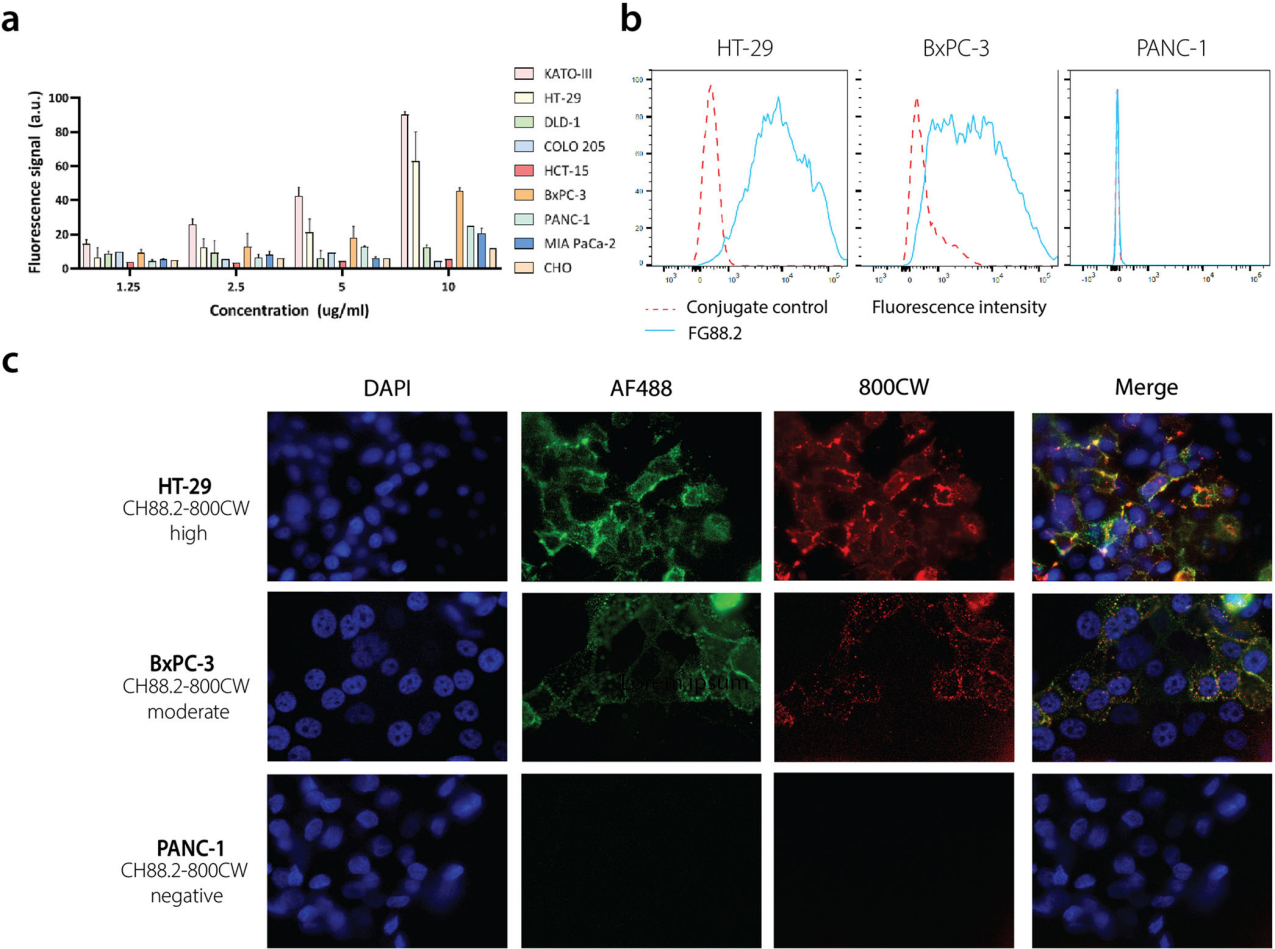
**a** Cell-based plate assay of FG88.2-800CW at 1.25, 2.5, 5, and 10 μg/ml dilutions on gastrointestinal cell lines. **b** Flow cytometry of FG88.2 on HT-29, BxPC-3, and PANC-1. Red-dotted lines represent conjugate controls and blue lines represent FG88.2 fluorescence signals. **c** Immunofluorescence analysis of CH88.2-800CW binding on HT-29, BxPC-3, and PANC-1 cells. AF488 signals and 800CW signals are represented in green and red, respectively. DAPI was used to stain nuclei (blue channel).

FG88.2 binding was evaluated on a panel of gastrointestinal carcinoma cell lines using a cell-based plate assay. CHO cells were included as a non-human negative control. Fluorescence signals increased in a dose-dependent manner and no relevant fluorescence signal was observed on CHO cells (Fig. 2a). High fluorescence signals were observed on KATO III and HT-29 cells, while fluorescence signal on BxPC-3 cells was moderate. Based on the observed fluorescence signals, HT-29 and BxPC-3 were selected as FG88.2-positive cancer cell lines for further studies and PANC-1 represented a low control. Next, FG88.2 binding to living cells was further confirmed using flow cytometry. HT-29, BxPC-3, and PANC-1 cells showed respectively high, moderate, and almost negative FG88.2 binding, in accordance with what was found using the plate assays (Fig. 2b). The binding specificity to these cells by the chimeric and NIRF conjugated counterpart CH88.2-800CW was performed in chamber slides using immunofluorescence. As expected, CH88.2-800CW expression (in red) was high on HT-29 cells, moderate on BxPC-3 cells, and not detectable on PANC-1 cells. Overlap of the 800CW signal with the AF488 signal (green), indicating the specific presence of anti-human antibodies, confirmed that the binding of 800CW-conjugated CH88.2 was specific (Fig. 2c). Based on these *in vitro* data, colon cancer HT-29 and pancreatic cancer BxPC-3 were selected as gastrointestinal cancer cell lines for *in vivo* binding studies with CH88.2-800CW,

### *In Vivo* NIRF Imaging of Subcutaneous HT-29 and BxPC-3 Tumors

**Fig. 3.**
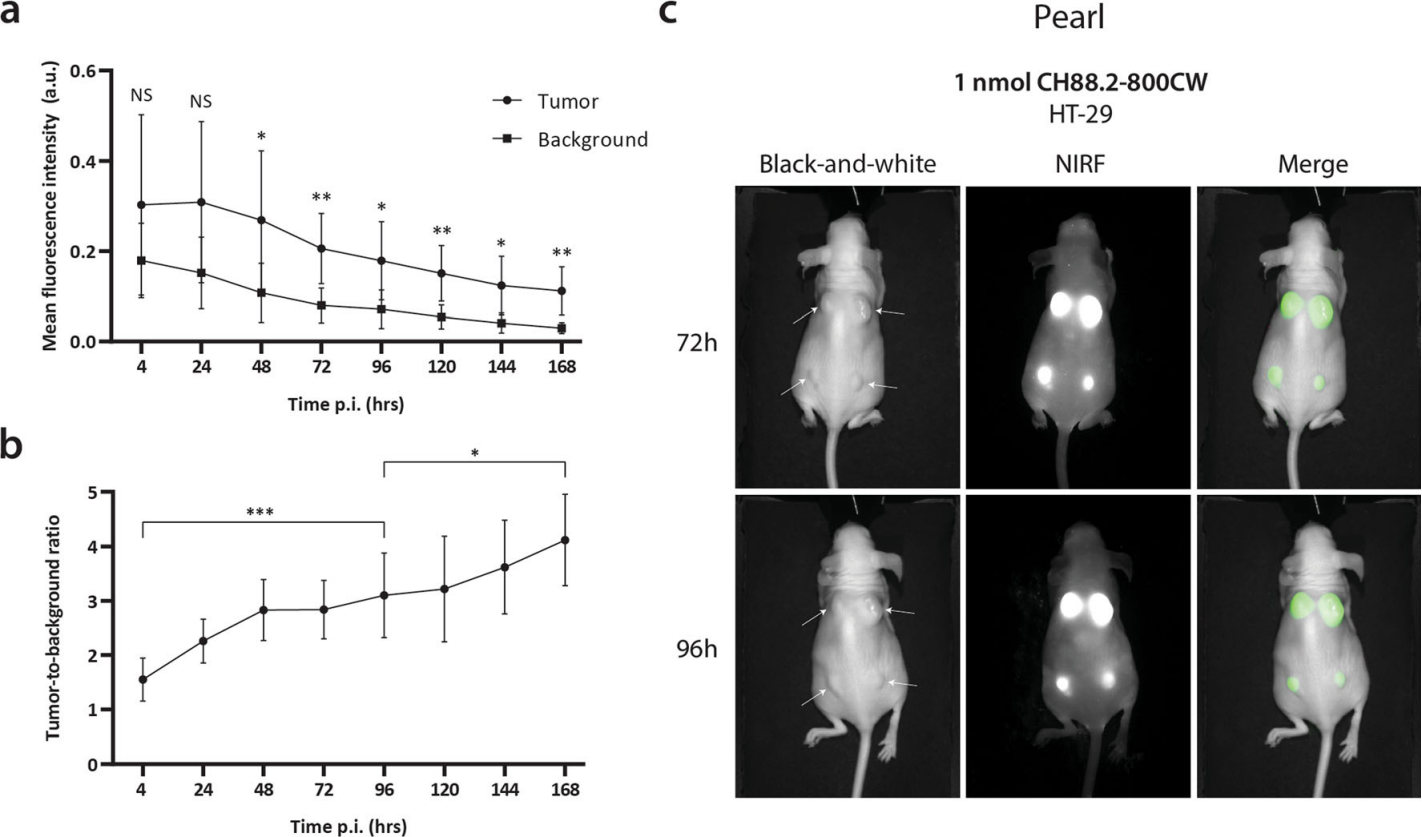
**a** Average tumor and background MFIs over time in HT-29 colon cancer-bearing mice injected with CH88.2-800CW using the Pearl preclinical imager. **b** Mean TBRs over time. **c** Representative black-and-white, NIRF, and merged images of HT-29 tumor-bearing mice at 72 h and 96 h post-injection.

**Fig. 4.**
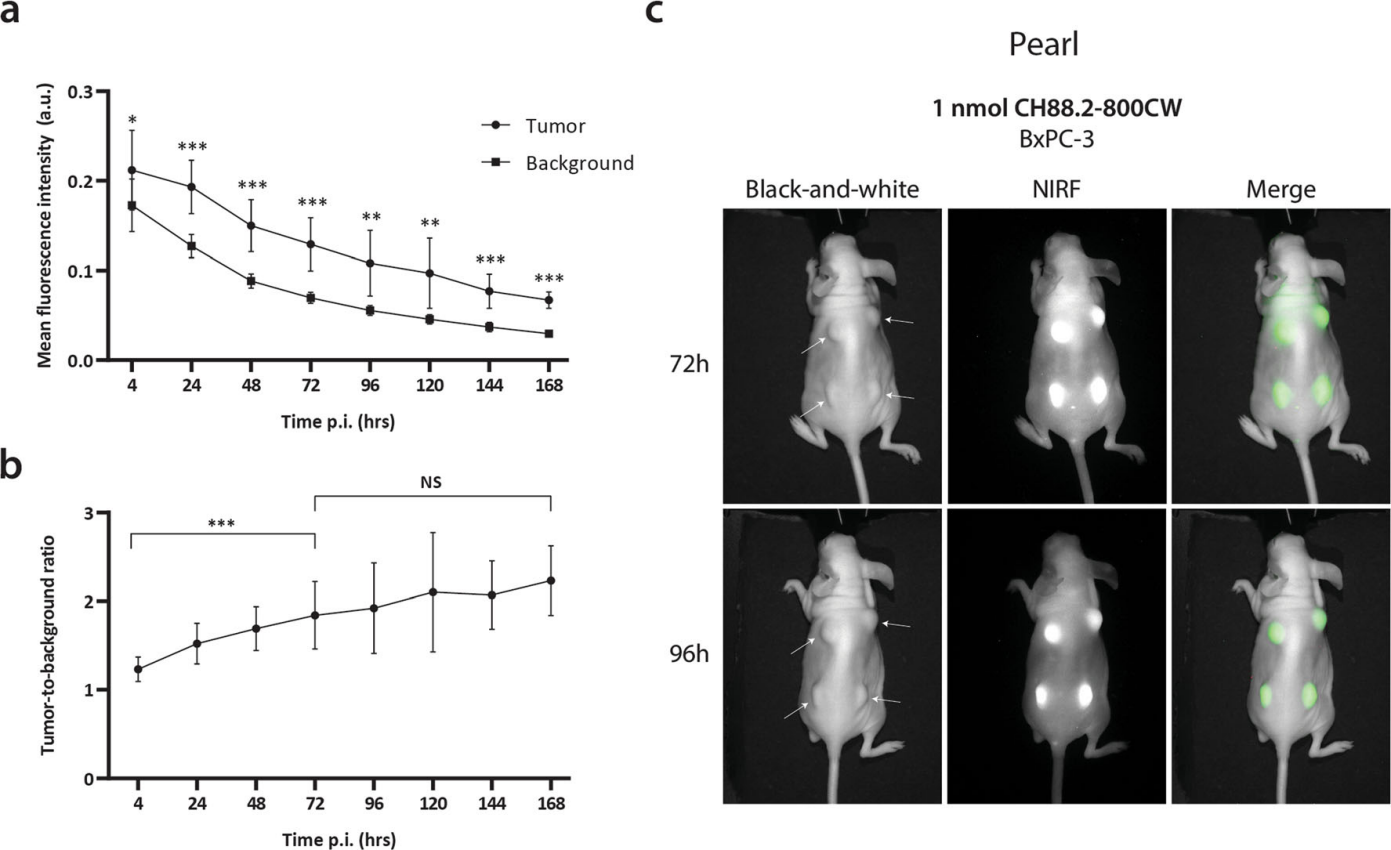
**a** Average tumor and background MFIs over time in BxPC-3 pancreatic cancer-bearing mice injected with CH88.2-800CW using the Pearl preclinical imager. **b** Mean TBR over time. **c** Representative black-and-white, NIRF, and merged images of BxPC-3 tumor-bearing mice at 72 h and 96 h post-injection.

To evaluate the *in vivo* binding of NIRF tracer CH88.2-800CW and establish the optimal imaging window, HT-29 and BxPC-3 tumor-bearing mice were injected with 1 nmol (150 μg) tracer and imaged every 24 h for 7 days (168 h) using the non-clinical Pearl imager. For the HT-29 colonic cancer model, significant differences between tumor and background MFIs could be detected from 48 h (*P* = 0.011) to 1 week (*P* = 0.003) and tumor MFIs were sufficient for tumor delineation at all time points onward (Fig. 3a). The optimal imaging time frame was defined at 96 h post-injection at which a TBR of 3.1 ± 0.8 was reached (Fig. 3b, c). TBRs continued to increase until 7 days post-injection (*P* = 0.017; Fig. 3b). Although the tumor MFI decreased over time, lesions could be clearly visualized during the optimal imaging window, which for many antibody-based tracers lies between 3 and 5 days post-injection [3]. In the BxPC-3 pancreatic cancer model, significant differences between tumor and background MFIs were observed as early as 4 h post-injection (*P* = 0.031) and remained significant until 168 h (*P* < 0.001; Fig. 4a). At the optimal imaging time point of 96 h post-injection, a TBR of 1.9 ± 0.5 was observed (Fig. 4b, c), which was sufficient to clearly localize all tumor lesions (Fig. 4c). Both gastrointestinal tumors could be clearly delineated up to 168 h post-injection (Suppl. Fig. 1, see ESM1).

**Fig. 5.**
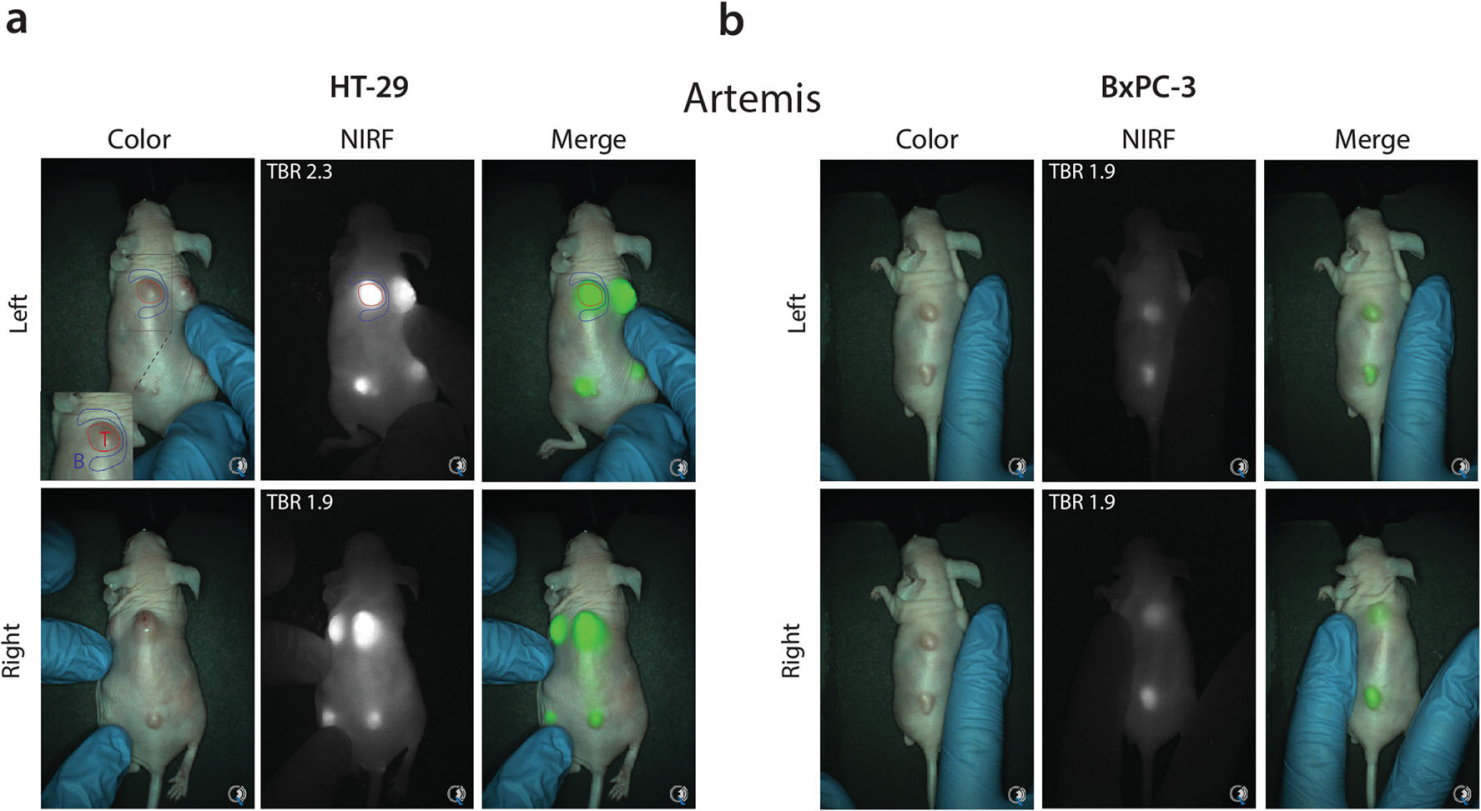
**a** Representative color, NIRF, and merged images of CH88.2-800CW binding specificity in a HT-29 tumor-bearing mouse model using the clinical Artemis NIR imaging system at 150-ms exposure. Regions of interest were selected in similar fashion to the Pearl as shown by the red and blue shapes, corresponding to the tumor and background area, respectively (only displayed in the left figure). To allow better visualization of the field of interest, the tumor-bearing skin was manually mobilized to the center of the camera’s optical field as is displayed by left and right back images. **b** Representative images of CH88.2-800CW binding specificity in a BxPC-3 tumor-bearing mouse model.

Next, NIRF imaging was performed using the clinically used Artemis NIR imaging system to highlight the translational potential of CH88.2-800CW-based tumor imaging. A clinical range exposure time of 150 ms was used, allowing real-time imaging. At the optimal imaging time point of 96 h, a mean TBR of 2.2 ± 0.3 was achieved in the HT-29 colonic model *versus* a mean TBR of 1.8 ± 0.3 in the BxPC-3 pancreatic model (Fig. 5, video clips available under ESM 2 and 3). Tumors could be localized and delineated excellently in both gastrointestinal cancer models up to 168 h post-injection (Suppl. Fig. 2, see ESM 1).

### *Ex Vivo* Imaging and Histological Analysis

**Fig. 6.**
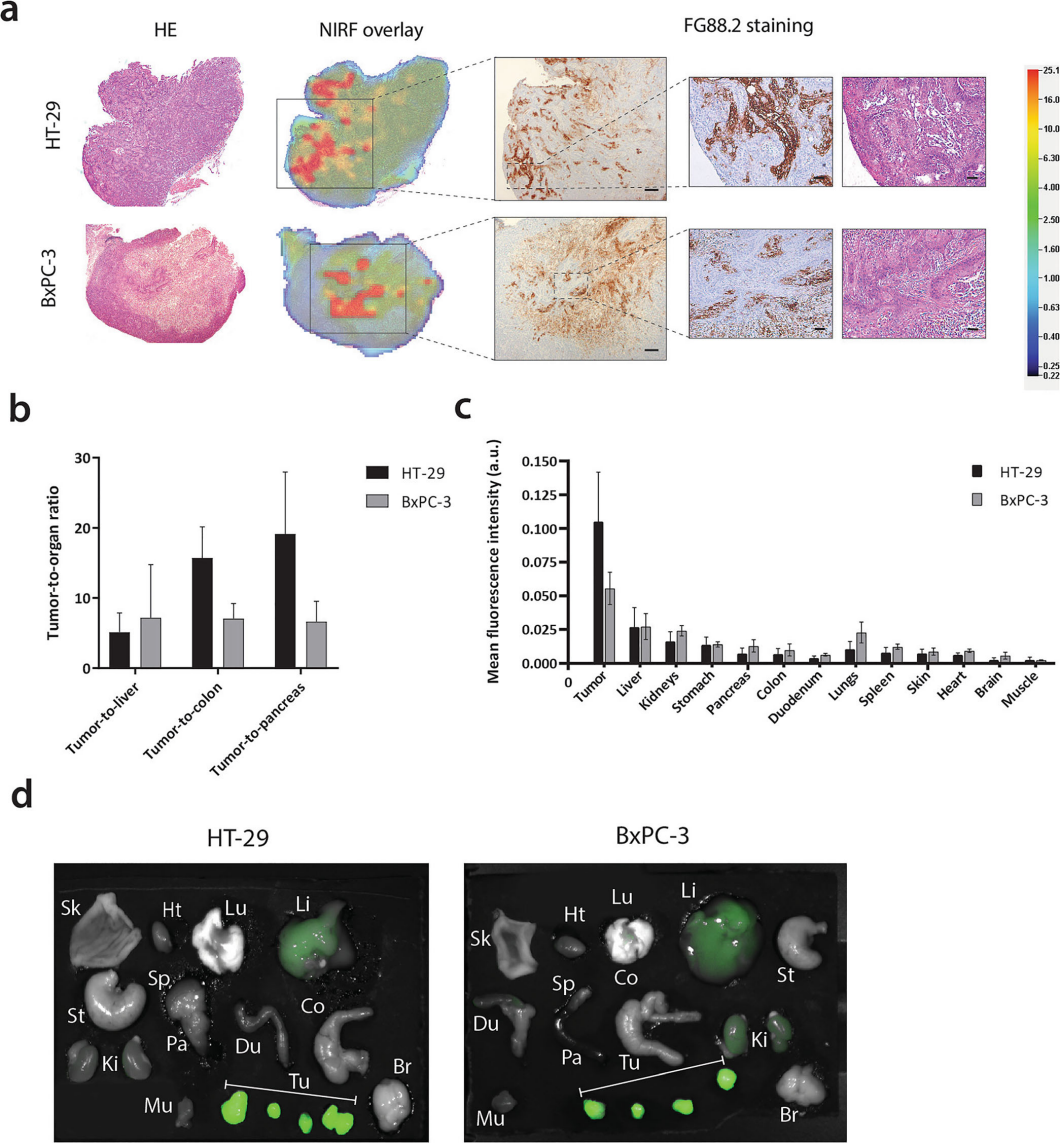
**a** Representative examples of *ex vivo* hematoxylin-eosin (HE) staining, NIR fluorescence heatmap (800 nm), and FG88.2 staining on HT-29 and BxPC-3 tumor tissue sections. Overview images are taken at × 25 magnification and inserts at × 100 magnification. Scale bars represent 500 μm and 100 μm for overview and insert images, respectively. **b** Average tumor-to-liver, tumor-to-colon, and tumor-to-pancreas ratios in HT-29 and BxPC-3 tumor-bearing mice at 168 h/1 week post-injection. **c** Biodistribution of CH88.2-800CW at 168h/1week post-injection expressed as tumor or organ MFI. **d***  Ex vivo* fluorescent images of resected tumors and organs. Sk, skin; Hrt, heart; Lu, lungs; Li, liver; St, stomach; Sp, spleen; Pa, pancreas; Du, duodenum; Co, colon; Ki, kidneys; Mu, muscle; Tu, tumors (under brackets); Br, brain.

At 1 week post-injection, mice were sacrificed and tumors were resected and sectioned. *Ex vivo* analysis showed that CH88.2-800CW fully penetrated the tumors, with a higher overall fluorescence signal in HT-29 colon tumors compared with BxPC-3 pancreatic tumors. FG88.2 staining on HT-29 and BxPC-3 tumors showed that Lewis^a/c/x^ was expressed in both models and expression correlated with the observed NIR signal (Fig. 6a). Of note, healthy mouse colon and pancreas tissues did show specific FG88.2 staining (Suppl. Fig. 5, see ESM 1).

Similarly, biodistribution of CH88.2-800CW at 1 week showed a high tumor uptake (HT-29: 0.105 ± 0.037; BxPC-3: 0.056 ± 0.012). High tumor-to-liver (HT-29: 5.1 ± 2.8; BxPC-3: 7.2 ± 7.6), tumor-to-colon (HT-29 15.7 ± 4.4; BxPC-3: 7.1 ± 2.2), and tumor-to-pancreas (HT-29: 19.1 ± 8.8; BxPC-3: 6.6 ± 2.9) ratios were achieved in both mouse models. Mean fluorescence signals in the organs associated with antibody clearing from the circulation were slightly higher than the other organs (liver: 0.027 ± 0.012 and kidneys: 0.020 ± 0.006). No statistically significant differences in biodistribution were observed between both mouse models (Fig. 6b, c).

## Discussion

In this study, we validated the concept of glycan-based tumor imaging, using a novel chimeric anti-Lewis glycan antibody, equipped with a clinically used NIRF dye. Although highest binding of FG88.2 was observed to KATO III cells, imaging of colorectal and pancreatic tumors was of particular interest considering their expression on well over 70 % of tumors. We showed that administration of CH88.2-800CW to human colon or pancreas tumor-bearing mice resulted in high-contrast tumor delineation using a clinical NIR camera system. Even though the target of FG88.2 is only moderately expressed on BxPC-3 cells, subcutaneous tumors could be localized within the optimal imaging window despite lower TBRs as found for HT-29. At 1 week post-injection of the tracer, tumor lesions could still be localized by the fluorescence signal. Imaging with CH88.2-800CW resulted in 2–3-fold higher TBRs than we have shown with 800CW or rituximab-800CW in the same HT-29 mouse model, suggesting specific binding of CH88.2 [4] (Baart et al., manuscript submitted). Full tumor penetration was confirmed using *ex vivo* analysis and tumor uptake seemed dependent on FG88.2 staining. Biodistribution of CH88.2-800CW showed the highly specific tracer uptake in both tumor types. Tumors could be easily delineated from healthy liver, colon, and pancreas tissues with high tumor-to-organ ratios. Compared with the low fluorescence signals in other organs, the liver and kidneys showed enhanced signals (< 50 % of tumor), which should be attributed to tracer clearance from the circulation. Our IHC results confirm the larger dataset previously published by Chua et al. and underscore the great *in vitro* and *in vivo* performance of the tracer for imaging of pancreatic, colon, and gastric carcinomas [18].

While surgery remains the cornerstone of cancer therapy, both untargeted and targeted FGS tracers have been implemented within standard-of-care in several centers, greatly affecting intraoperative decision-making through identification of tumor tissue and visually occult lesions [2, 8, 21]. By using a chimeric monoclonal antibody (mAb) and a clinically available dye and camera system, we have demonstrated the great translational potential of CH88.2-800CW for NIRF imaging of gastrointestinal tumors. A possible limitation of the current study is that TBRs may have been overestimated as mice do not naturally express Le^a/c/x^ glycans, which was supported by our IHC results [22]. Another limitation of the study is that we did not evaluate Le^a/c/x^ expression on precursor lesions, tumor-positive lymph nodes, and metastases, which should also be distinguished from surrounding tissues. Although we have reported lower FG88.2 staining in normal human tissues than in their malignant tissue counterparts, a more detailed IHC analysis of FG88.2 is essential to establish the potential and specific employability of CH88.2-800CW for tumor imaging.

Monoclonal antibody FG88.2 binds the Le^c^Le^x^-glycan and Le^c^Le^x^-related glycan clusters, as well as the single Le^a^ subunit. Le^a^ overexpression has been observed in the majority of gastrointestinal cancers [23–28] and gastric lesions, such as gastritis and intestinal metaplasia, suggesting a potential role for CH88.2-800CW in early gastric cancer detection [29]. Additionally, Le^a^ expression has been observed in chronic pancreatitis and loss of Le^a^ expression was observed in colonic polyps [25, 30]. Therefore, our observation that pancreatitis tissues did not stain for FG88.2 is encouraging a clinical application, since the distinction between pancreatitis and tumor tissue poses a major challenge during surgery for pancreatic cancer. To a lesser extent, Le^a^ is also expressed in several normal tissues such as normal pancreas, distal colon, and stomach, which may explain the mild FG88.2 reactivity with these human tissues [26, 28, 31]. Although in principle a non-tumor reactivity can hamper the suitability of a tumor imaging tracer, the ratio of expression between tumor and adjacent normal tissue (TBR) seems to be well over two for most organs, including the lungs. Besides, the limited expression of FG88.2 on normal tissues was largely confined to the apical membrane and it is unlikely that circulating antibodies will reach these locations *in vivo* [18]. Noteworthy, FGS of human colon tumors targeting the EpCAM glycoprotein resulted in excellent tumor localization despite relatively low TBRs of around two [5].

Tumor-associated glycans are of particular interest in the quest for novel, less conventional targets for improved tumor imaging. Several preclinical and clinical studies validated anti-Lewis glycan antibodies for therapy or imaging, particularly focusing on sLe^a^, also known as CA19-9. Preclinically, administration of anti-CA19-9 antibody HuMab-5B1 doubled survival time of COLO 205 (colon carcinoma) tumor-bearing mice and, remarkably, resulted in full survival of two mice at a higher dose without toxicity [32]. The NIRF dye- and or ^89^Zr-labeled HuMab-5B1 mAb variants were also validated for PET imaging and FGS, with excellent tumor delineation, resection, metastasis imaging, and sentinel lymph node mapping possibilities in both a subcutaneous and orthotopic model of pancreatic cancer [33, 34]. Phase I trials validating HuMab-5B1 for PET imaging (NCT02687230), radioimmunotherapy (NCT03118349), and immunotherapy (NCT02672917) in pancreatic cancer and other CA19-9 expressing malignancies are currently active or recruiting in the USA. Although sLe^a^ is highly expressed in > 90 % of pancreatic cancers, it is also overexpressed in normal pancreatic tissue and chronic pancreatitis. Furthermore, sLe^a^ serum levels are elevated in benign diseases such as pancreatitis, cholangitis, and obstructive jaundice, all making the distinction between cancer and non-cancerous pancreaticobiliary diseases potentially challenging when targeting CA19.9 alone [35, 36]. Thus, Lewis glycan-based tumor imaging seems feasible, but using alternative Lewis glycans, such as Le^a/c/x^, which are not expressed by normal and benign tissues, may pave the way for an even more specific and/or broader tumor-targeting strategy.

Altogether, our proof-of-concept study demonstrates the potential of imaging gastrointestinal tumors by targeting Lewis glyco-epitopes present on cancer cells with the novel, NIR dye-conjugated chimeric monoclonal antibody CH88.2-800CW. As the tracer consists of a chimeric mAb and a FDA-approved NIR fluorescent dye, it is ready for clinical use, making a rapid clinical translation by our group feasible [5, 9, 10].

## Conclusions

To conclude, our results show that both colorectal and pancreatic tumors can be excellently delineated after administration of Lewis glycan-specific CH88.2-800CW, with low tracer uptake in other tissues. This promising proof-of-concept research not only paves the way for a more extensive evaluation of the CH88.2-800CW tracer for FGS but also demonstrates the relevance of glycans for real-time imaging of gastrointestinal tumors. By conducting this study, we form a firm foundation for the introduction of glycan-targeted molecular imaging to the operating room of the future.

## Electronic Supplementary Material

ESM 1 (DOCX 1.90 mb)

ESM 2 (AVI 18.2 mb)

ESM 3 (AVI 18.2 mb)

## References

[1] Verbeek FP, van der Vorst JR, Schaafsma BE (2012). Image-guided hepatopancreatobiliary surgery using near-infrared fluorescent light. J Hepatobiliary Pancreat Sci.

[2] Hernot S, van Manen L, Debie P, Mieog JSD, Vahrmeijer AL (2019). Latest developments in molecular tracers for fluorescence image-guided cancer surgery. Lancet Oncol.

[3] Boonstra MC, Tolner B, Schaafsma BE, Boogerd LSF, Prevoo HAJM, Bhavsar G, Kuppen PJK, Sier CFM, Bonsing BA, Frangioni JV, van de Velde CJH, Chester KA, Vahrmeijer AL (2015). Preclinical evaluation of a novel CEA-targeting near-infrared fluorescent tracer delineating colorectal and pancreatic tumors. Int J Cancer.

[4] van Driel PBAA, Boonstra MC, Prevoo HAJM, van de Giessen M, Snoeks TJA, Tummers QRJG, Keereweer S, Cordfunke RA, Fish A, van Eendenburg JDH, Lelieveldt BPF, Dijkstra J, van de Velde CJH, Kuppen PJK, Vahrmeijer AL, Löwik CWGM, Sier CFM (2016). EpCAM as multi-tumour target for near-infrared fluorescence guided surgery. BMC Cancer.

[5] Boogerd LSF, Boonstra MC, Prevoo H (2019). Fluorescence-guided tumor detection with a novel anti-EpCAM targeted antibody fragment: preclinical validation. Surg Oncol.

[6] Dijkers EC, Oude Munnink TH, Kosterink JG, Brouwers AH, Jager PL, de Jong JR, van Dongen GA, Schröder CP, Lub-de Hooge MN, de Vries EG (2010). Biodistribution of 89Zr-trastuzumab and PET imaging of HER2-positive lesions in patients with metastatic breast cancer. Clin Pharmacol Ther.

[7] Handgraaf HJM, Boonstra MC, Prevoo H (2017). Real-time near-infrared fluorescence imaging using cRGD-ZW800-1 for intraoperative visualization of multiple cancer types. Oncotarget.

[8] Boogerd LSF, Hoogstins CES, Schaap DP, Kusters M, Handgraaf HJM, van der Valk MJM, Hilling DE, Holman FA, Peeters KCMJ, Mieog JSD, van de Velde CJH, Farina-Sarasqueta A, van Lijnschoten I, Framery B, Pèlegrin A, Gutowski M, Nienhuijs SW, de Hingh IHJT, Nieuwenhuijzen GAP, Rutten HJT, Cailler F, Burggraaf J, Vahrmeijer AL (2018). Safety and effectiveness of SGM-101, a fluorescent antibody targeting carcinoembryonic antigen, for intraoperative detection of colorectal cancer: a dose-escalation pilot study. Lancet Gastroenterol Hepatol.

[9] Rosenthal EL, Warram JM, de Boer E, Chung TK, Korb ML, Brandwein-Gensler M, Strong TV, Schmalbach CE, Morlandt AB, Agarwal G, Hartman YE, Carroll WR, Richman JS, Clemons LK, Nabell LM, Zinn KR (2015). Safety and tumor specificity of cetuximab-IRDye800 for surgical navigation in head and neck cancer. Clin Cancer Res.

[10] Lamberts LE, Koch M, de Jong JS, Adams ALL, Glatz J, Kranendonk MEG, Terwisscha van Scheltinga AGT, Jansen L, de Vries J, Lub-de Hooge MN, Schröder CP, Jorritsma-Smit A, Linssen MD, de Boer E, van der Vegt B, Nagengast WB, Elias SG, Oliveira S, Witkamp AJ, Mali WPTM, van der Wall E, van Diest PJ, de Vries EGE, Ntziachristos V, van Dam GM (2017). Tumor-specific uptake of fluorescent bevacizumab-IRDye800CW microdosing in patients with primary breast cancer: a phase I feasibility study. Clin Cancer Res.

[11] Pinho SS, Reis CA (2015). Glycosylation in cancer: mechanisms and clinical implications. Nat Rev Cancer.

[12] Munkley J, Elliott DJ (2016). Hallmarks of glycosylation in cancer. Oncotarget.

[13] Pinho S, Marcos NT, Ferreira B, Carvalho AS, Oliveira MJ, Santos-Silva F, Harduin-Lepers A, Reis CA (2007). Biological significance of cancer-associated sialyl-Tn antigen: modulation of malignant phenotype in gastric carcinoma cells. Cancer Lett.

[14] Munkley J (2016). The role of Sialyl-Tn in Cancer. Int J Mol Sci.

[15] Blanas A, Sahasrabudhe NM, Rodríguez E, van Kooyk Y, van Vliet SJ (2018) Fucosylated antigens in cancer: an alliance toward tumor progression, metastasis, and resistance to chemotherapy. Frontiers in oncology, 8, 39 10.3389/fonc.2018.00039 PMC5829055 29527514

[16] Fuster MM, Esko JD (2005). The sweet and sour of cancer: glycans as novel therapeutic targets. Nat Rev Cancer.

[17] Rabu C, McIntosh R, Jurasova Z, Durrant L (2012). Glycans as targets for therapeutic antitumor antibodies. Future Oncol.

[18] Chua JX, Vankemmelbeke M, McIntosh RS (2015). Monoclonal antibodies targeting LecLex-related glycans with potent antitumor activity. Clin Cancer Res.

[19] Mahler Convenor M, Berard M, Feinstein R (2014). FELASA recommendations for the health monitoring of mouse, rat, hamster, guinea pig and rabbit colonies in breeding and experimental units. Lab Anim.

[20] Rueden CT, Schindelin J, Hiner MC, DeZonia BE, Walter AE, Arena ET, Eliceiri KW (2017). ImageJ2: ImageJ for the next generation of scientific image data. BMC Bioinformatics.

[21] van Manen L, Handgraaf HJM, Diana M, Dijkstra J, Ishizawa T, Vahrmeijer AL, Mieog JSD (2018). A practical guide for the use of indocyanine green and methylene blue in fluorescence-guided abdominal surgery. J Surg Oncol.

[22] Gersten KM, Natsuka S, Trinchera M, Petryniak B, Kelly RJ, Hiraiwa N, Jenkins NA, Gilbert DJ, Copeland NG, Lowe JB (1995). Molecular cloning, expression, chromosomal assignment, and tissue-specific expression of a murine alpha-(1,3)-fucosyltransferase locus corresponding to the human ELAM-1 ligand fucosyl transferase. J Biol Chem.

[23] Tauchi K, Kakudo K, Machimura T, Makuuchi H, Mitomi T (1991). Immunohistochemical studies of blood group-related antigens in human superficial esophageal carcinomas. Cancer.

[24] Pour PM, Tempero MM, Takasaki H (1988). Expression of blood group-related antigens ABH, Lewis A, Lewis B, Lewis X, Lewis Y, and CA 19-9 in pancreatic cancer cells in comparison with the patient’s blood group type. Cancer Res.

[25] Itzkowitz SH, Yuan M, Ferrell LD, Ratcliffe RM, Chung YS, Satake K, Umeyama K, Jones RT, Kim YS (1987). Cancer-associated alterations of blood group antigen expression in the human pancreas2. J Natl Cancer Inst.

[26] Itzkowitz SH, Yuan M, Fukushi Y, Lee H, Shi ZR, Zurawski V Jr, Hakomori S, Kim YS (1988). Immunohistochemical comparison of Lea, monosialosyl Lea (CA 19-9), and disialosyl Lea antigens in human colorectal and pancreatic tissues. Cancer Res.

[27] Schoentag R, Primus FJ, Kuhns W (1987). ABH and Lewis blood group expression in colorectal carcinoma. Cancer Res.

[28] Sakamoto S, Watanabe T, Tokumaru T (1989). Expression of Lewisa, Lewisb, Lewisx, Lewisy, siayl-Lewisa, and sialyl-Lewisx blood group antigens in human gastric carcinoma and in normal gastric tissue. Cancer Res.

[29] Serpa J, Mesquita P, Mendes N, Oliveira C, Almeida R, Santos-Silva F, Reis CA, LePendu J, David L (2006). Expression of Lea in gastric cancer cell lines depends on FUT3 expression regulated by promoter methylation. Cancer Lett.

[30] Itzkowitz SH, Yuan M, Ferrell LD, Palekar A, Kim YS (1986). Cancer-associated alterations of blood group antigen expression in human colorectal polyps. Cancer Res.

[31] Blaszczyk M, Pak KY, Herlyn M, Sears HF, Steplewski Z (1985). Characterization of Lewis antigens in normal colon and gastrointestinal adenocarcinomas. Proc Natl Acad Sci U S A.

[32] Sawada R, Sun SM, Wu X, Hong F, Ragupathi G, Livingston PO, Scholz WW (2011). Human monoclonal antibodies to sialyl-Lewis (CA19.9) with potent CDC, ADCC, and antitumor activity. Clin Cancer Res.

[33] Houghton JL, Zeglis BM, Abdel-Atti D, Aggeler R, Sawada R, Agnew BJ, Scholz WW, Lewis JS (2015). Site-specifically labeled CA19.9-targeted immunoconjugates for the PET, NIRF, and multimodal PET/NIRF imaging of pancreatic cancer. Proc Natl Acad Sci U S A.

[34] Houghton JL, Abdel-Atti DA, Sawada R, Scholz WW, Lewis JS (2015). Abstract B25: development of 5B1, an anti-CA19.9 monoclonal antibody, as a near-infrared fluorescent probe for intraoperative imaging of pancreatic cancer. Cancer Res.

[35] Ballehaninna UK, Chamberlain RS (2011). Serum CA 19-9 as a biomarker for pancreatic cancer—a comprehensive review. Indian journal of surgical oncology.

[36] Schwenk J, Makovitzky J (1989). Tissue expression of the cancer-associated antigens CA 19-9 and CA-50 in chronic pancreatitis and pancreatic carcinoma. Int J Pancreatol.

