# Supplementary material for: Glycan-Based Near-infrared Fluorescent (NIRF) Imaging of Gastrointestinal Tumors: a Preclinical Proof-of-Concept In Vivo Study
Source: Mol Imaging Biol. 2020 Aug 11;22(6):1511–22. doi: 10.1007/s11307-020-01522-8 (PMC7666282; doi:10.1007/s11307-020-01522-8)
Supplement: Supplementary file 1 — (DOCX 1.90 mb) [file 11307_2020_1522_MOESM1_ESM.docx]

**ELECTRONIC SUPPLEMENTARY MATERIAL 1**

**Title**

Glycan-based near-infrared fluorescent (NIRF) imaging of gastrointestinal tumors: a preclinical proof-of-concept *in vivo* study.

**Authors**

Ruben D. Houvast^1*^, Victor M. Baart^1*^, Shadhvi S. Bhairosingh^1^, Robert A. Cordfunke^2^, Jia Xin Chua^3^, Mireille Vankemmelbeke^3^, Tina Parsons^3^, Peter J. K. Kuppen^1^, Lindy G. Durrant^3,4^, Alexander L. Vahrmeijer,^1^, Cornelis F. M. Sier^1,5^

**Author affiliations**

^1^Department of Surgery, Leiden University Medical Centre, Leiden, the Netherlands

^2^Department of Immunohematology and Blood Transfusion, Leiden University Medical Centre, Leiden, the Netherlands

^3^Scancell Limited, University of Nottingham Biodiscovery Institute, University Park, Nottingham, UK

^4^Division of Cancer and Stem Cells, School of Medicine, University of Nottingham Biodiscovery Institute, University Park, Nottingham, UK

^5^Percuros BV, Leiden, the Netherlands

*Both authors contributed equally to this work and share first-authorship.

**Corresponding author:**  Dr. Cornelis F.M. Sier

Albinusdreef 2, 2333 ZA Leiden, The Netherlands

Phone: +31752662610

Fax: +31752666750

E-mail: c.f.m.sier@lumc.nl

**
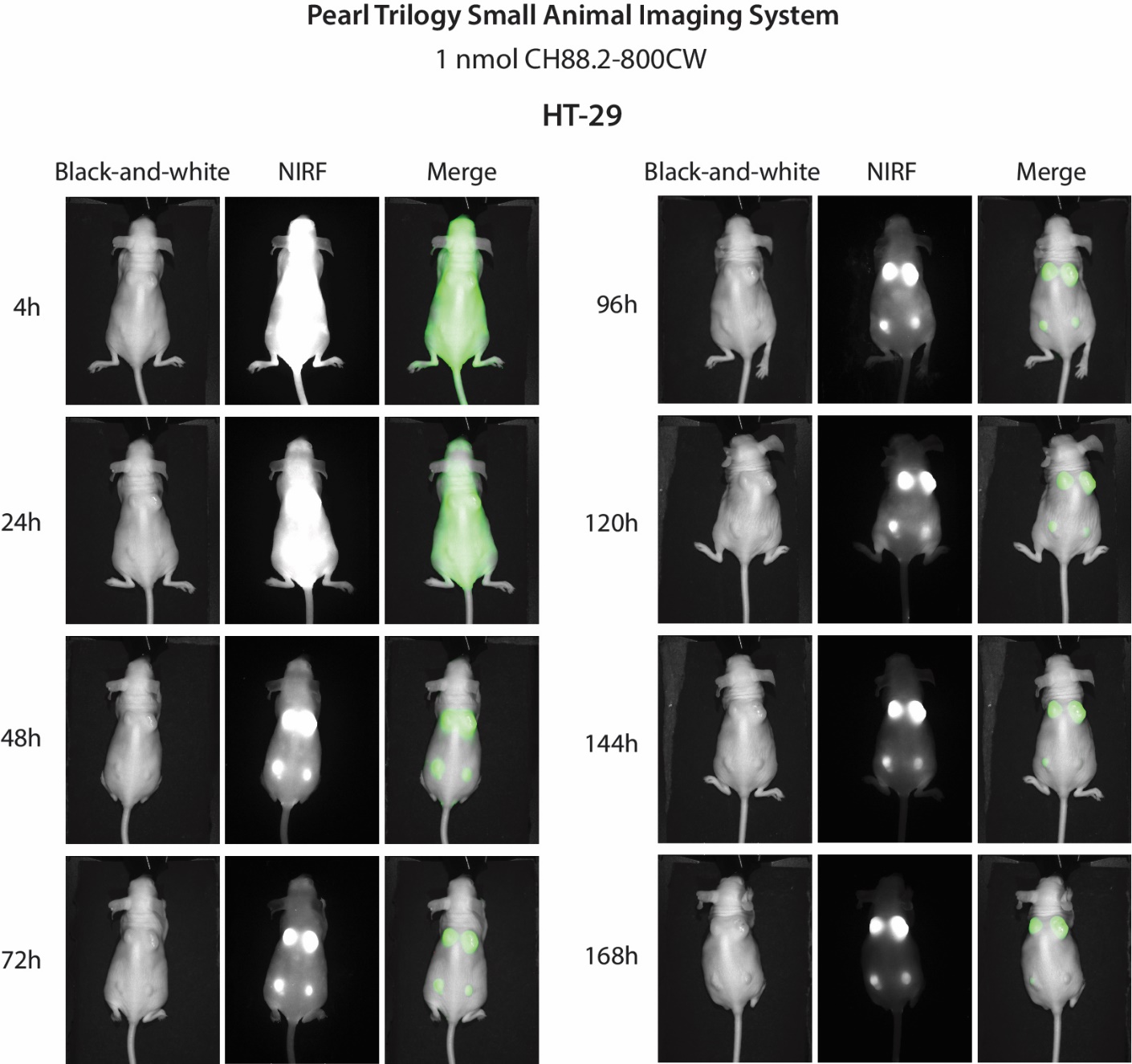
Supplementary Figure 1** *In vivo* images at all time points of HT-29 colon cancer-bearing mice using the Pearl preclinical imager.


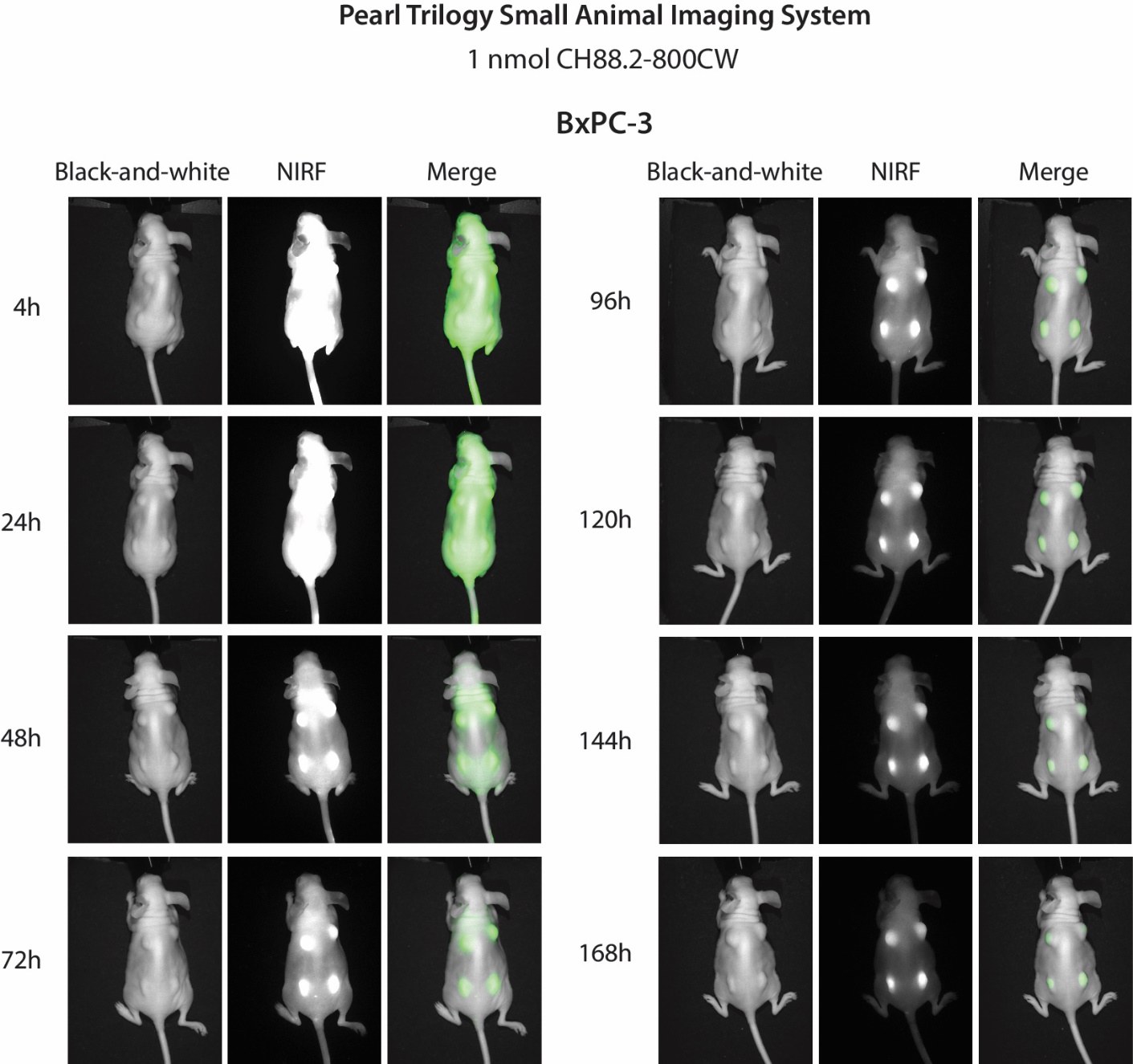
**Supplementary Figure 2** *In vivo* images at all time points of BxPC-3 pancreatic cancer-bearing mice using the Pearl preclinical imager.


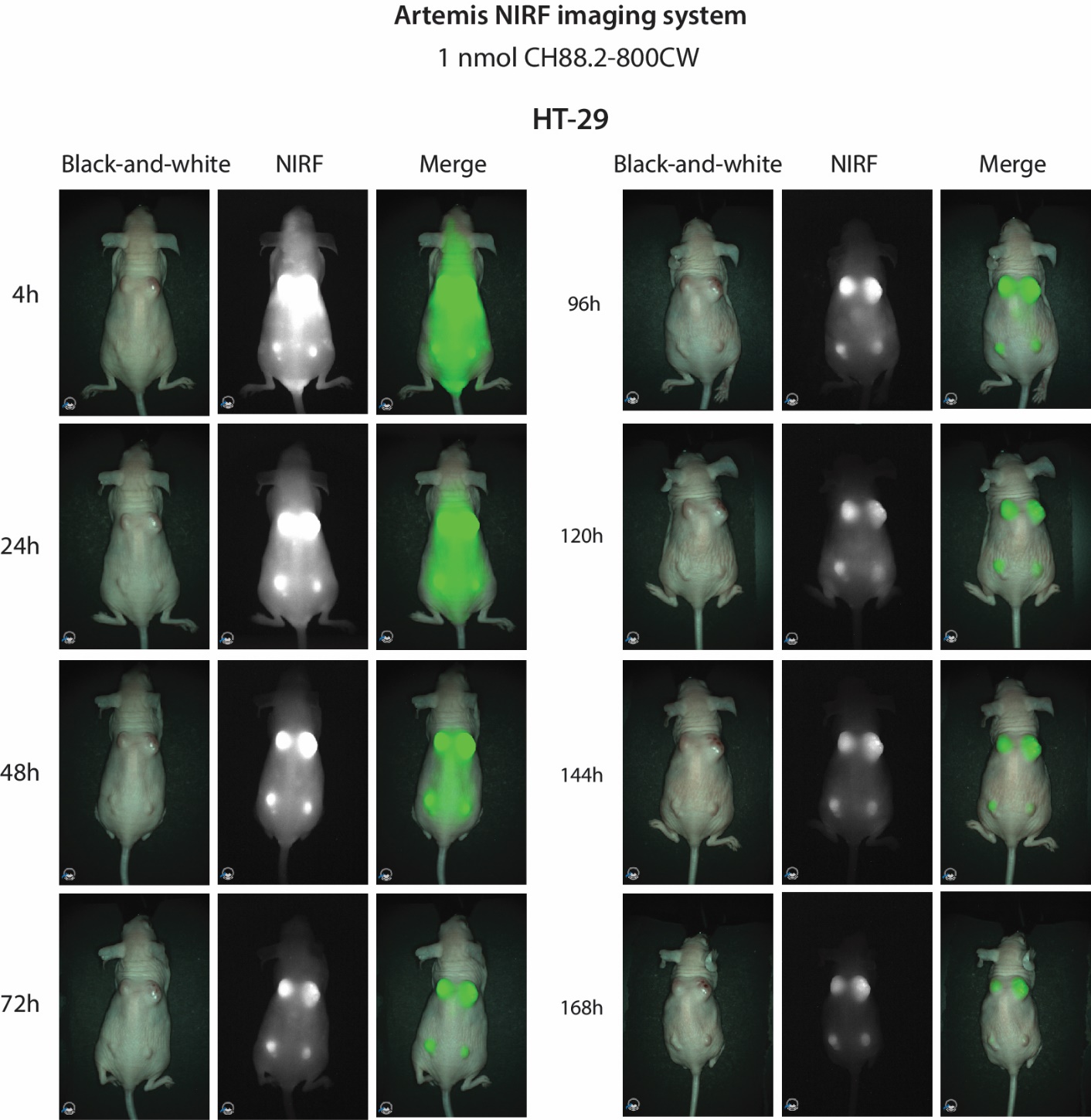
**Supplementary Figure 3** *In vivo* images at all time points of HT-29 colon cancer-bearing mice using the clinical Artemis imager.

**
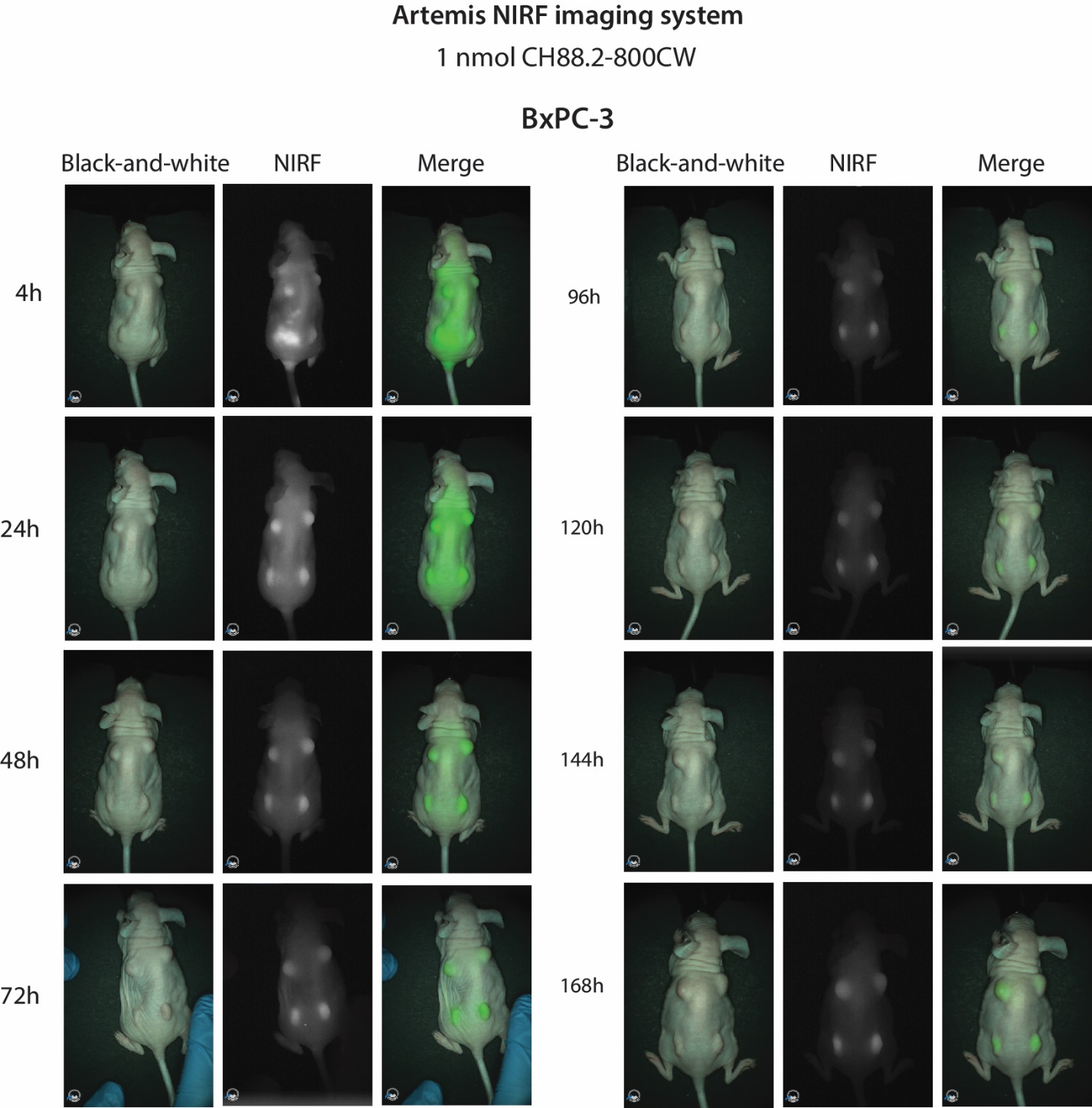
Supplementary Figure 4** *In vivo* images at all time points of BxPC-3 pancreatic cancer-bearing mice using the clinical Artemis imager.


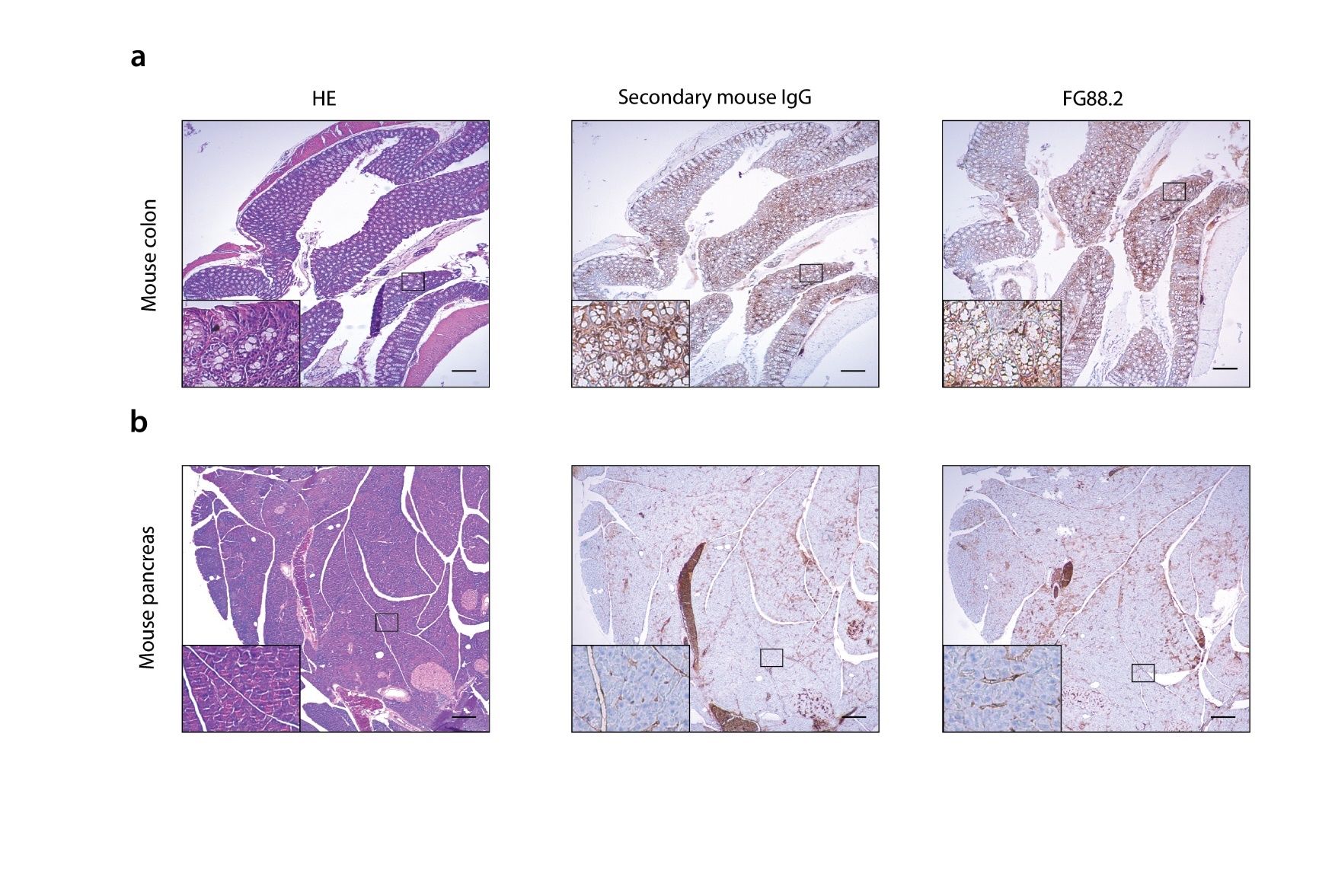
.

**Supplementary Figure 5** HE, secondary mouse IgG (conjugate control) and FG88.2 staining on **a** healthy mouse colon and **b** normal mouse pancreas tissues. Similar staining patterns were observed for secondary mouse IgG and FG88.2, suggesting that mouse colon and pancreas do not express Le^a/c/x^-related glycans. Overview images are taken at x 25 magnification and scale bars represent 500 µm.

**HE**
